# Reclassification of Inflammatory Bowel Disease Type Unclassified by Small Bowel Capsule Endoscopy

**DOI:** 10.3390/medicina59122064

**Published:** 2023-11-23

**Authors:** Ana-Maria Singeap, Catalin Sfarti, Irina Girleanu, Laura Huiban, Cristina Muzica, Sergiu Timofeiov, Carol Stanciu, Anca Trifan

**Affiliations:** 1 Department of Gastroenterology, Faculty of Medicine, “Grigore T. Popa” University of Medicine and Pharmacy, 700115 Iasi, Romania; anamaria.singeap@yahoo.com (A.-M.S.); gilda_iri25@yahoo.com (I.G.); huiban.laura@yahoo.com (L.H.); lungu.christina@yahoo.com (C.M.); stanciucarol@yahoo.com (C.S.); ancatrifan@yahoo.com (A.T.); 2 Institute of Gastroenterology, “St. Spiridon” University Hospital, 700111 Iasi, Romania; 3 Department of Surgery, Faculty of Medicine, “Grigore T. Popa” University of Medicine and Pharmacy, 700115 Iasi, Romania; 4 Department of Surgery, “St. Spiridon” University Hospital, 700111 Iasi, Romania

**Keywords:** inflammatory bowel disease type unclassified, Crohn’s disease, ulcerative colitis, small bowel capsule endoscopy

## Abstract

*Background and Objectives*: Ulcerative colitis (UC) and Crohn’s disease (CD) are idiopathic inflammatory bowel diseases (IBDs) without a unique, gold standard diagnostic test. UC and Crohn’s colitis are impossible to distinguish in approximately 10% of cases. The term IBD type unclassified (IBD-U) is recommended for cases of chronic colitis showing overlapping endoscopic, radiological, and biopsy histological features between UC and CD, while indetermined colitis is reserved for colectomy specimens. Our aim was to assess the role of small-bowel capsule endoscopy (SBCE) in the diagnostic work-up of IBD-U. *Materials and Methods*: We retrospectively studied the cases of IBD-U explored by SBCE in a tertiary referral gastroenterology center. Patients were investigated using SBCE after contraindications were excluded. Diagnostic criteria for small bowel CD consisted in more than three ulcerations, irregular ulcers, or stenosis, and the Lewis score was used for the quantification of inflammation. The immediate impact of reclassification and outcome data was recorded over a follow-up period of more than one year. *Results*: Twenty-eight patients with IBD-U were examined using SBCE. Nine patients had small bowel lesions that met the diagnostic criteria for CD, resulting in a reclassification rate of 32.1%. In five of these cases, the treatment was subsequently changed. In the remaining nineteen examinations, no significant findings were observed. There were no complications associated with SBCE. Median follow-up time was 32.5 months (range 12–60). During follow-up, twelve patients were classified as having UC, and seven remained as having an unclassified type; one case of colectomy, for medically refractory UC, was recorded. *Conclusions*: SBCE is a useful safe tool in the work-up of IBD-U, allowing reclassification in about one third of cases, with subsequent treatment modifications. SBCE may provide a definite diagnosis, enhance the comprehension of the disease’s progression, and optimize the short- and long-term management strategy.

## 1. Introduction

Ulcerative colitis (UC) and Crohn’s disease (CD) are complex and invalidating chronic inflammatory bowel diseases (IBDs), with partially understood development mechanisms. They result from a combination of genetic, immunological, and environmental factors [1]. These diseases can manifest as various phenotypes, distinguished by unique features such as localization, extent, progression, and the likelihood of developing different complications; however, generally, while there are often shared clinical features among IBD patients, they are not identical. Each individual, whether suffering from UC or CD, will exhibit a distinctive combination of symptoms. Therefore, a tailored disease assessment is essential to establishing an accurate diagnosis. A unique, gold standard test for IBD diagnosis is currently unavailable. The diagnosis relies on a combination of clinical elements—symptoms, history, and physical exam, as well as biological, endoscopic, ultrasound, radiological, and histological findings [2]. In some cases, reassessment after several months may be necessary to confirm the chronic nature of the inflammatory condition. Furthermore, there are instances where the presentation of UC and Crohn’s colitis is so similar that in 4–10% of cases, they cannot be distinguished [3,4]. The term IBD unclassified (IBD-U) is recommended for the cases of chronic colitis where endoscopic, radiological, and biopsy histological features do not reveal a clear distinction between UC and CD, while indetermined colitis is reserved for colectomy specimen [5,6]. The lack of a definite diagnosis may have implications for patient management ant outcome. Since small-bowel involvement can be observed exclusively in Crohn’s disease (CD), it is essential to employ accurate non-invasive methods for exploring the small bowel to ensure the most effective approach in such cases. In this context, we aimed to assess the role of small bowel capsule endoscopy (SBCE) in the diagnostic work-up of IBD-U.

## 2. Materials and Methods

We conducted a retrospective analysis of all cases of IBD-U that underwent SBCE within a five-year period, from 1st of August 2017 to 31st of July 2022, at a tertiary referral gastroenterology center. This study received approval from the local Ethical Committee and adhered to the principles outlined in the Declaration of Helsinki. Informed consent was obtained from all subjects involved in the study.

### 2.1. Patients

All the patients included in the study presented with chronic colitis that exhibited overlapping features of both UC and CD. Despite undergoing ileocolonoscopy with multiple mucosal biopsies and histological examinations, a definitive diagnosis had not been established. These patients had a minimum disease history of six months, during which they had previously undergone appropriate investigations, either via computed tomography enterography (CTE) or magnetic resonance enterography (MRE). In all cases, an infective cause had been conclusively ruled out and a comprehensive work-up failed to identify any other underlying cause of colitis.

### 2.2. Small Bowel Capsule Endoscopy

Patients were investigated by SBCE after the thorough exclusion of contraindications. The exclusion criteria were as follows: known or suspected stricture, obstructive symptoms at the time of evaluation or in the past, swallowing disorders, the presence of a pace-maker or other implantable electromedical device, and pregnancy. A comprehensive clinical assessment, which included patient history, physical examination, and imaging data, was conducted to identify those patients at risk of capsule retention. The previous administration of nonsteroidal anti-inflammatory drugs was meticulously documented, and patients underwent the SBCE procedure only after a minimum four-week period without any nonsteroidal anti-inflammatory drugs use. Preparation involved the ingestion of two liters of polyethylene glycol on the day preceding the procedure; the capsule was ingested in the morning following an overnight fast. The third generation of small bowel capsule endoscopy (PillCam SB3, manufactured by Given Imaging, Yoqneam, Israel) was utilized. Video analysis was performed using the Rapid Reader software versions 8 and 9. The interpretation of images was carried out by an experienced gastroenterologist, who had specialized training in capsule endoscopy. Capsule endoscopy structured terminology, as originally proposed by Korman et al. [7], has been used to describe the various lesions identified during the SBCE procedure. The diagnostic criteria for CD were based on Mow’s criteria—consisting of the presence of more than three ulcerations, the absence of nonsteroidal anti-inflammatory drugs [8], or the presence of irregular ulcers or stenosis. Mucosal breaks and erosions were not considered diagnostic for CD; a minimum size of 0.5 cm was considered suggestive for diagnosis. For patients fulfilling these criteria, the Lewis score was assessed [9]. The Lewis score divides the small bowel into tertiles (proximal, middle, and distal), and disease severity was determined by evaluating the following three endoscopic criteria: villous edema, ulceration, and stenosis. The overall score was derived from the sum of the worst-affected tertile in terms of edema und ulceration, plus the score of stenoses, which were evaluated considering the entire length of the small bowel. A score below the threshold of 135 is typically regarded as normal or clinically insignificant, scores that fall within the range of 135 to 790 points are indicative of mild inflammation, while a score that surpasses 790 points indicates the presence of moderate-to-severe inflammation [9]. This score is incorporated into the reading software used for analysis. If the predetermined criteria for CD were met, patients were subsequently reclassified as having CD. If no features of CD were identified in the small bowel during SBCE examination, the patients retained their original unclassified status. Complications of SBCE procedure, especially, though not limited to capsule retention, were assessed.

### 2.3. Follow-Up Data

Changes in the treatment and outcome data were recorded for all patients, for at least one year following the SBCE examination.

### 2.4. Statistical Analysis

The analysis in this study primarily involved descriptive statistics to characterize the data. The following statistical measures were utilized: median, mean, standard deviation, and percentages. The median was used to represent variables as follow-up time, the mean (average) was calculated for age and Lewis score, standard deviation was used to assess the dispersion of data points around the mean, and percentages were utilized to express the categorical data, such as the patients’ gender, the proportion of reclassified patients, or patients with changes in management.

## 3. Results

A total of twenty-eight patients who were diagnosed with unclassified colitis were examined using SBCE, over a five-year period, as detailed in Table 1. There were no patients with swallowing disorders. None of the patients presented a pacemaker or other implantable electromedical device. The study did not include pregnant women.

Criteria consistent with the diagnosis of CD were found in nine patients, resulting in a reclassification rate of 32.1%. SBCE identified multiple ulcers distributed across one, two, or all three segments of the small bowel; additionally, other findings included erosions, edema, and hyperemia, which are detailed in Table 2.

According to the Lewis score, it was determined that six patients exhibited mild inflammation, while three patients presented with moderate-to-severe inflammation. Among the patients who were reclassified following SBCE examination, changes in treatment plans were implemented in a total of five cases (the three patients with moderate-to-severe inflammation, and other two patients with mild small bowel inflammation). This accounts for 55.5% of the patients reclassified as having CD, and 17.8% from all cases of IBD-U that underwent SBCE, respectively. The modifications in treatment primarily involved the discontinuation of 5-aminosalycilic derivatives and the initiation of different therapeutic strategies. Specifically, in three cases, azathioprine was introduced, while the remaining two cases required the implementation of biologic therapy.

Of the remaining nineteen examinations, none revealed findings within the small bowel. During the follow-up period, ten of these patients were reclassified, with their diagnoses being updated to UC. The remaining nine patients retained their initial unclassified status. One case of colectomy for medically refractory disease was recorded in the group of patients with unclassified colitis. In the postoperative assessment, the colectomy specimen analysis confirmed the diagnosis as UC. Following colectomy, this patient’s condition exhibited a favorable evolution.

No complications due to the SBCE examination occurred.

## 4. Discussion

According to the existing literature, up to 10% of colonic IBD patients defy straightforward classification as either CD or UC based solely on colonoscopy and histological findings [3,4]. Moreover, clinical studies have shed light on the dynamic nature of these conditions. Over the course of their illness, around 3% of patients initially diagnosed with UC have undergone reclassification, being identified as having CD. On the other hand, a range of 0.6% to 3% in patients who initially received a CD diagnosis have been subsequently reclassified as having UC [10], underscoring the evolving and complex diagnostic landscape in the realm of IBD.

The term IBD-U is conventionally employed to describe cases of chronic colitis in which a comprehensive evaluation, including endoscopic, radiological, and histological biopsy findings, fails to provide a clear-cut distinction between UC and CD. On the other hand, “indeterminate colitis” is specifically reserved for cases involving colectomy specimens, where, despite an in-depth examination, the precise nature of the colitis cannot be definitively ascertained [5,6].

Debates continue to persist within the medical literature regarding whether IBD-U truly represents a distinct and unique phenotype of IBD, or if it reflects the inherent diagnostic challenges posed by several factors, including atypical onset, a variable clinical presentation, inconclusive results from imaging studies, or a lack of definitive histological features. This issue is particularly pronounced in the pediatric population, where unclassified colitis accounts for a higher proportion, potentially reaching up to 30% [11,12]. The unresolved issue is whether IBD-U indeed constitutes a distinct and separate disease entity or simply reflects the inherent complexities in characterizing a definitive phenotype, particularly in the very early stages of life.

Assigning the label of IBD-U to a patient’s condition typically necessitates a prior evaluation of the small bowel. This evaluation is conventionally carried out through non-invasive radiological techniques, such as MRE, CTE, or intestinal ultrasound.

MRE is recognized as having high accuracy in diagnosing CD, making it a non-invasive and highly effective method for assessing both small bowel involvement and potential intestinal or extraintestinal complications [13]. However, it is important to note that MRE has limitations, as it is not able to directly visualize the small bowel mucosa, which accounts for its relatively lower sensitivity in detecting early lesions associated with CD in comparison to SBCE [14]. Furthermore, SBCE has demonstrated its superiority over MRE in identifying inflammatory lesions located in the proximal segment of the small intestine [15]. Nonetheless, MRE remains a valuable option for ongoing disease monitoring.

CTE is another diagnostic option for detecting CD. However, it is essential to emphasize that, in addition to not exhibiting superiority over MRE or SBCE [16], its effectiveness is hindered by the considerable drawback of ionizing radiation exposure.

Intestinal ultrasound (IUS) is a rapidly gaining recognition as a non-invasive diagnostic technique, featuring several inherent strengths including widespread accessibility and a strong safety profile [17]. Due to its capacity to detect intestinal inflammation, achieved through the measurement of bowel wall thickness, the detection of changes in bowel stratification, increased vascularization, and the description of extramural features, IUS is a very helpful method for IBD diagnosis. Studies showed good accuracy of IUS in the diagnosis of CD, with overall sensitivity ranging from 54% to 93%, and a remarkable high specificity ranging between 97% and 100% [18]. IUS is most valuable monitoring tool in established CD cases as it facilitates the assessment of IBD activity and the close monitoring of the patient’s response to therapeutic interventions [19].

In real-world clinical practice, patients initially classified as having IBD-U frequently undergo a process of reclassification to CD upon the discovery of small bowel involvement, which was previously unknown [3]. The patients in our study had previously undergone diagnostic assessment, either through CTE or MRE, failing to reveal any definitive findings that strongly suggested a diagnosis of CD. Following these initial investigations, they were subsequently referred for SBCE.

SBCE is a non-invasive diagnostic method that is typically recommended as the first-line investigation for suspected small bowel pathology, provided there are no contraindications [20]. One of its primary advantages lies in its unique capability to directly visualize the entire small bowel mucosa. This comprehensive examination allows for the identification of small-bowel lesions suggestive for CD, ultimately facilitating the reclassification of some patients initially categorized as IBD-U. While enteroscopy offers the advantage of histological analysis, it is an invasive procedure that fails short in entirely visualizing the small bowel. Consequently, the data provided by SBCE, particularly concerning the presence and location of lesions, are often considered a prerequisite for guiding the insertion route during enteroscopy, emphasizing the role played by SBCE in the diagnostic pathway.

The rates of reclassification present variability across different studies, ranging from 16% to 44% [4,21,22], depending on the choice of diagnostic method and the characteristics of the study population. Although not validated, Mow’s criteria, consisting in the presence of more than three ulcerations in the absence of nonsteroidal anti-inflammatory drugs, have gained widespread acceptance for diagnosing CD [8]. Due to its capability to detect early ulcerative lesions, SBCE allows reclassification in a higher number of cases than non-endoscopic imaging techniques [23]. Additionally, it seems that the proportion of reclassification is higher when SBCE is used as a discriminative method during a flare of the underlying inflammatory disease [24]. Considering cases that were reclassified with a diagnosis of CD following SBCE, according to Mow’s criteria, we found a reclassification rate of 32.1%. All the patients displayed more than three ulcerations, which were distributed across one, two, or all three small bowel tertiles. A minimum size of 0.5 cm was considered sufficient for diagnosis. Erosions and mucosal breaks were not regarded as diagnostic criteria, given their potential occurrence in up to 14% of healthy individuals [25]. Nevertheless, the utilization of non-validated diagnostic criteria could be considered a limitation of our study. Moreover, we also documented additional findings, such as mucosal hyperemia, villous edema, villous denudations, erosions, or mucosal breaks. None of the patients enrolled in our study underwent nonsteroidal anti-inflammatory drug administration during at least four weeks prior to the examination, mitigating potential confounding factors.

Several scoring systems have been developed to quantitatively assess the small bowel inflammation, as the Lewis score [9] and the Capsule Endoscopy Crohn Disease Activity Index [26]; despite their reciprocal correlation, these scoring systems tend to exhibit only limited correlation with clinical and laboratory parameters [27,28]. However, mucosal inflammation, as evaluated through capsule scores, plays a prediction role in poor outcomes. This role positions it as a valuable tool to guide the treatment intensification [29]. Two thirds of the patients in our analysis who met Mow’s criteria had mild inflammation scores—likely corresponding to early subtle lesions, while one-third of the patients displayed higher scores, requiring subsequent changes in their treatment plans.

In general, the lesions described by SBCE must be interpreted with caution, given that capsule endoscopy relies on a visual inspection and lacks the capacity to obtain tissue biopsies. When a diagnosis is uncertain, histological examination becomes necessary, which can be facilitated through device-assisted enteroscopy. However, the patients in our study had previously received a diagnosis of colonic IBD, and exhaustive evaluations had excluded other colonic pathologies. Despite the indistinguishable phenotypes of UC and CD, other colonic pathologies had been effectively ruled out.

Although a negative SBCE cannot definitively rule out the possibility of CD, our follow-up data suggest that in the absence of SB lesions, considering these cases to be UC is a reasonable approach. Treating them accordingly appears to correlate with a favorable outcome. During the follow-up period, none of the patients was subsequently reclassified as CD. However, ten out of the nineteen patients with negative SBCE results were later reclassified as UC. There was a single exception of refractory UC that ultimately required colectomy. Importantly, this outcome was attributed to the natural course of UC evolution rather than the diagnostic process. Furthermore, postoperative histological analysis consistently confirmed the diagnosis of UC.

Nonetheless, a future diagnosis of CD cannot be entirely ruled out based on a normal SBCE [30]. Given that the ultimate diagnosis is generally established within the initial eight years of disease’s development [31], the consideration of a second SBCE examination may be deemed appropriate in cases where clinical indication arise during long-term follow-up. A large retrospective study involving 44,302 patients showed that changes in the IBD subtype occurred in 18% of cases during a median follow-up period of 3.8 years, while in the subgroup of IBD-U patients, a change in diagnosis was noted in 67% of cases [32]. Since all our patients had a relatively short disease history, SBCE emerges as a rapid and effective discrimination method for accurately categorizing their conditions.

The clinical impact of reclassification may involve treatment modifications, including escalation, the introduction of new medications, or the adjustment of monitoring strategies to align with the updated diagnosis. Small bowel involvement carries an inherent risk for future complications. Therefore, achieving a clear and precise IBD diagnosis essential is imperative for ensuring appropriate and effective long-term follow-up.

Patients diagnosed with unclassified colitis typically receive a management approach that closely resembles the one applied to patients with UC [33]. Medical treatment consists of aminosalicylates, corticosteroids, thiopurines, biologics, calcineurin inhibitors, and small molecules. The treatment strategy for IBD-U is determined by various factors, including the clinical severity of the disease, as well some endoscopic and histological particularities. While aminosalicylates remain the foremost treatment option for inducing and maintaining remission in patients with mild to moderate UC, their effectiveness in the management of CD has not been conclusively established. It is essential to highlight that there is a lack of specific trial data to provide strong evidence regarding the aminosalicylate derivatives in the context of IBD-U. Nevertheless, in the everyday clinical setting, their utilization in the treatment of unclassified colitis is a fairly common practice [34]. However, aminosalicylates have not demonstrated any advantages in either the induction or maintenance of remission in the case of Crohn’s disease, and as such, they are not recommended for the treatment of CD. Even so, in the real world, many CD patients are prescribed aminosalicylates at some point during the course of their disease. In our study, aminosalicylates were discontinued in the majority of patients who were reclassified as having CD, with the option of immunosuppressive treatment. However, choices exhibited variability, as they were shaped by the unique clinical characteristics and individualized clinical judgement.

In cases of suspected CD, studies have reported a risk of capsule retention of approximately 3%, whereas for patients with an established diagnosis of CD, this risk escalates to around 8% [35,36]. In our analysis, retention did not occur. This is likely attributable to the careful selection of patients at risk, who were not referred for SBCE. The structuring phenotype of CD usually manifests obstructive symptoms or present with suggestive imaging data, both of which are reasons to avoid SBCE. Moreover, the majority of cases examined through SBCE in our study displayed mild to moderate inflammatory lesions, without evidence of small bowel stenosis, further contributing to the absence of capsule retention incidents. However, when deciding to perform SBCE, caution is warranted due to the retention risk. Nevertheless, the utilization of a patency capsule can serve as an additional safety measure.

Further research is necessary to gain a more comprehensive understanding of the natural history of IBD-U. While some cases of IBD-U may arise from initial misdiagnosis during the early stages or incomplete diagnostic investigations, others present unique challenges due to their atypical phenotypes. Although in certain cases, reclassification may not significantly impact the management of the condition, there are situations, particular when surgical interventions are considered, where achieving the right classification becomes paramount. This is particularly crucial given that the rate of complications is notably higher in CD [37]. The insights provided by capsule endoscopy studies may have the potential to enhance our understanding of the disease, offering valuable information concerning its localization, extension, and severity.

## 5. Conclusions

SBCE stands out as a valuable and safe tool in the assessment of IBD-U, allowing reclassification in approximately one-third of cases, and prompting subsequent adjustments to the treatment plan. Thanks to its diagnostic capabilities, SBCE can provide a definitive diagnosis and also contributes to an improved understanding of the disease’s progression. Although clinical decisions vary due to a multitude of individual factors, the insights offered by capsule endoscopy concerning small bowel involvement are invaluable. SBCE should be included in the diagnostic repertoire along with alternative investigations to ensure a comprehensive assessment. SBCE can be a cornerstone in refining IBD patients’ approach and optimizing both short- and long-term management strategies.

## Figures and Tables

**Table 1:** Clinical characteristics of the IBD-U patients and outcome after SBCE.

	Patients, Total Number = 28
Females, *n* (%) Males, *n* (%)	10 (35.7%) 18 (64.3%)
Age, years (mean ± SD, range)	34.2 ± 14.5 (19–61)
Disease history, months (range)	6–28
Reclassified as CD, *n* (%) Lewis score, points (mean ± SD, range) Treatment change, *n* (%)	9 (32.1%) 484 ± 236 (180–876) 5 (55.5%)
Follow-up after SBCE, months (median, range)	32.5 (12–60)
Capsule retention, *n*	0
Reclassified during FU As CD As UC	10 0 10
Colectomy during FU, *n* Colectomy among reclassified patients, *n*	1 0

IBD-U: inflammatory bowel disease type unclassified; SBCE: small bowel capsule endoscopy; FU: follow-up; CD: Crohn’s disease; UC: ulcerative colitis.

**Table 2:** SBCE findings allowing reclassification.

Patients’ Findings Consistent with CD		Additional Findings
1.	Four ulcerations in the second tertile	Patchy hyperemia Diffuse villous edema Erosions Patchy villous denudation Mucosal breaks
2.	More than eight ulcerations, along the entire SB length	
3.	More than eight ulcerations, in the first and second tertile	
4.	Five ulcerations in the second and third tertile	
5.	Four ulcerations in the third tertile	
6.	More than eight ulcerations, along the entire SB length	
7.	Four ulcerations in the first and second tertile	
8.	Five ulcerations, in the first, second, and third tertile	
9.	More than eight ulcerations, along the entire SB length	

## Data Availability

The data presented in this study are available on request from the corresponding author.
